# Label Free Fragment Screening Using Surface Plasmon Resonance as a Tool for Fragment Finding – Analyzing Parkin, a Difficult CNS Target

**DOI:** 10.1371/journal.pone.0066879

**Published:** 2013-07-05

**Authors:** Karin Regnström, Jiangli Yan, Lan Nguyen, Kari Callaway, Yanli Yang, Linnea Diep, Weimei Xing, Anirban Adhikari, Paul Beroza, Roy K. Hom, Brigit Riley, Don Rudolph, Michael F. Jobling, Jeanne Baker, Jennifer Johnston, Andrei Konradi, Michael P. Bova, Rick D. Artis

**Affiliations:** Elan Pharmaceuticals, South San Francisco, California, United States of America; Spanish National Cancer Center, Spain

## Abstract

Surface Plasmon Resonance (SPR) is rarely used as a primary High-throughput Screening (HTS) tool in fragment-based approaches. With SPR instruments becoming increasingly high-throughput it is now possible to use SPR as a primary tool for fragment finding. SPR becomes, therefore, a valuable tool in the screening of difficult targets such as the ubiquitin E3 ligase Parkin. As a prerequisite for the screen, a large number of SPR tests were performed to characterize and validate the active form of Parkin. A set of compounds was designed and used to define optimal SPR assay conditions for this fragment screen. Using these conditions, more than 5000 pre-selected fragments from our in-house library were screened for binding to Parkin. Additionally, all fragments were simultaneously screened for binding to two off target proteins to exclude promiscuous binding compounds. A low hit rate was observed that is in line with hit rates usually obtained by other HTS screening assays. All hits were further tested in dose responses on the target protein by SPR for confirmation before channeling the hits into Nuclear Magnetic Resonance (NMR) and other hit-confirmation assays.

## Introduction

The ubiquitin-proteasome system (UPS) is central to a variety of different cellular events, and its dysregulation could be a contributing factor to the pathogenesis of neurodegenerative diseases such as Alzheimer's disease (AD), Parkinson's disease (PD) and Amyotrophic Lateral Sclerosis (ALS). The inhibition or activation of targets involved in the UPS pathway has proved to be a difficult area for drug discovery and drug development partially due to a lack of good functional assays for screening Currently, only one drug targeting the UPS pathway has been approved by the Food and Drug Administration, an inhibitor of the 26S-proteasome subunit with an anticancer indication [1]. UPS signaling is mediated by the covalent linkage of multiple units of ubiquitin to lysine residues of proteins. Ubiquitin is attached to proteins by the interplay of three enzymes, an E1 activating enzyme, an E2 conjugating enzyme and an E3 ubiquitin ligase. As E3 ubiquitin ligases are the last step in the regulatory enzyme cascade, they are an attractive drug target. However, the lack of robust and well defined high-throughput screening (HTS) assays for E3 ubiquitin ligases has been a significant barrier to the discovery of agonists/antagonists [2]. Currently, no direct binding screens or fragment screens for E3 ubiquitin ligases have been reported in the literature.

Parkin is linked to Familial Parkinson's Disease (FPD) by mutations in PARK2 on chromosome 6q25.2–27 and is abundantly expressed in brain and a variety of tissues [3]. Parkin is an E3 ubiquitin ligase, and its function involves the transfer of ubiquitin (Ubq) from Conjugating Enzyme E2 to substrates through attachment to ε-amino atoms of lysines or nitrogen atoms at the N-terminus. Several mechanisms of ubiquitin transfer have been proposed [4], and a large variety of substrates has been identified [5]. Ubiquitination of substrates leads to their proteosomal degradation, signaling events or formation of inclusions [3]. Loss of Parkin's E3 ligase activity, and therefore loss of ubiquitination of proteins, has been linked to neurodegeneration. In patients harboring Parkin mutations, a selective loss of dopaminergic neurons in the substantia nigra has been observed. Therefore, it is highly desirable to identify a small molecule agonist that is capable of stabilizing functionally active Parkin.

**Figure 1:**
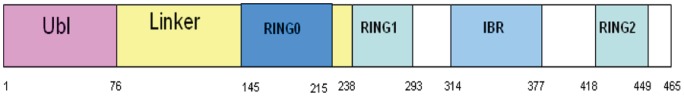
Schematic representation of Parkin domains.

Parkin consists of 465 amino acids and has a molecular mass of 51.65 kDa. Parkin is a difficult protein to screen; it is known to easily aggregate [6] and contains 35 cysteines and 8 Zinc atoms (7), which are required for structural stability and enzymatic activity [7]. Several models have been proposed for the coordination of the zinc atoms [7]. Parkin comprises several domains: the Ubiquitin-like domain (UblD), a linker domain, three Ring domains R0, R1 and R2, and an IBR domain between R1 and R2 (see Figure 1). All three Ring domains coordinate two zinc atoms each, and an additional two zinc atoms are found in the IBR domain [7]. The UblD structure is very similar to ubiquitin and differs only in the C-terminal region when both structures are superimposed [8]. It has been proposed that Parkin can exist in an auto-inhibited state in which the UblD domain is folded back and binds to a Parkin UblD-Ubq binding (PUB) motif between the IBR domain and Ring2 domain. In this state Parkin cannot be auto-ubiquitinated. Binding of activators to Parkin may result in conformational changes that reveal motifs involved in substrate binding and charged E2-interaction [9]. Consistent with this possibility, N-terminal tags to full-length Parkin (FL-Parkin) have been reported to increase auto-ubiquitination [10].

Previous HTS screens for small molecules binding to E3 ligases have utilized enzymatic activity assays [11] or biochemical assay approaches, such as *in vitro* ubiquitination assay [2], [12]. Our previous Parkin HTS was based on such an ubiquitination assay and resulted in the discovery of a variety of chemical scaffolds, which formed the basis of SAR efforts (data not shown). However, hit expansion efforts did not markedly improve potency or increase residency times (see Figure S2). Previously, a successful direct binding assay screen has not been reported in the literature. Herein, SPR was used to characterize the E3 ligase Parkin, to perform assay development for optimization of screening conditions, and to identify new scaffolds that bind to Parkin from a fragment screening campaign. This is, to our knowledge, the first fragment screen performed on an E3 ligase using SPR as the primary screening technology.

## Materials and Methods

CM5 chips, amine coupling kit, HBS-P Buffer (10 mM HEPES pH 7.4, 100 mM NaCl, 0.005% Tween-20), Tween-20 solution were purchased from GE Healthcare (Piscataway, NJ), Glutathione-S Transferase (GST) and Carbonic Anhydrase II (CA) was obtained from R&D Systems (Minneapolis, MN). S5a, Ubiquitin and UbcH7 were purchased from Boston Biochem (Cambridge, MA) and dimethyl sulfoxide (DMSO), pluronic F127 detergent, Anti-FLAG antibody M2 from Sigma Aldrich (Rockville, MD). Streptavidin XL665 conjugate and Ub-Eu K were obtained from Cisbio (Bedford, MA). Filter plates and vacuum device were obtained from Millipore Corporation (Billerica, MA) and 96- or 384 well plates from Greiner Bio-One Inc. (Monroe, NC). High performance Ni sepharose, the Mono Q HR 10/10 anion exchange column, and the HiLoad 26/60 Superdex 200 column were from GE Life Sciences. The Anti-FLAG M2 agarose resin and the 3x-FLAG peptide were obtained from Sigma Aldrich. 10% Bis-Tris Nu PAGE gels and MES running Buffer were from Life Technologies.

### Constructs and protein expression

To generate the FLAG-Parkin expression construct, the entire coding region (residues 1–465) from NM_004562.1 was cloned into pFastBac (Invitrogen) with an N-terminal 3xFLAG and tobacco etch virus (TEV) protease cleavage site (MDYKDHDGDYKD HDIDYKDDDDKENLYFQSS). Recombinant Bacmid was generated and P1 virus was produced and amplified according to the manufacturer's protocol (Invitrogen). For protein production, the amplified virus was used to infect either Sf9 or Sf21 cells; pellets were harvested 2–3 days post infection by centrifugation and snap frozen in liquid nitrogen prior to purification.

To generate the R0-RBR expression construct, DNA encoding residues 141–465 of Parkin (NM_004562.1) were cloned into the Champion pET SUMO vector (Invitrogen) according to the manufacturer's protocol. To generate the Ubch7 expression construct, DNA encoding residues 1–154 of UBE2L3 (NM_003347.2) was cloned into the pET30 vector with an N terminal 8xHis/TEV site (MHHHHHHHHENLYFQSS). To generate the Ubl expression construct, DNA encoding residues 1–76 of Parkin (NM_004562.1) was cloned into the pET30 vector incorporating a C-terminal 6xHis tag followed by two stop codons. Bacterial expression of R0-RBR, Ubch7, and Ubl was as follows: plasmids were transformed into BL21 DE3 *E.Coli* (Invitrogen). Overnight cultures inoculated from fresh colonies were grown in Terrific broth media containing 2% glucose and 50 μg/ml kanamycin at 37°C. The following morning overnight cultures were diluted to OD_600_ 0.1 and shaking was continued at 37°C until OD_600_ reached 0.4, at which time the flasks were transferred to 16°. When the OD_600_ reached 0.8 to 0.9, cultures were induced with 0.1 mM IPTG supplemented with 50 uM zinc chloride (R0-RBR only) and expression was allowed to proceed for 18–20 hours at 16°C. Cells were then harvested by centrifugation and frozen at −80°C.

### Protein purification

#### FLAG-Parkin

Frozen pellets were resuspended in buffer A (50 mM Tris pH 7.4, 150 mM NaCl, and Complete protease inhibitor tablets) and lysed using a microfluidizer. The lysate was cleared (45,000 g, 25 min, 4°C) and the supernatant agitated gently with Anti-FLAG M2 agarose (12.5 ml resin/L cell culture) for 1 hr at 4°C. The beads were washed with 10 column volumes of buffer A and the Parkin protein was eluted with 10 column volumes of buffer A containing 100 µg/ml 3X FLAG peptide. After elution, 1mM TCEP was added and the protein was diluted with 50 mM Tris to reduce the salt concentration to 20 mM NaCl. The protein was then loaded onto a Mono Q HR 10/10 anion exchange column that had been pre-equilibrated in buffer B (50 mM Tris pH 7.4 and 1 mM TCEP). The column was developed with a gradient of 0−500 mM NaCl over 34 column volumes, and the protein was eluted at 100–140 mM NaCl. Collected fractions were then concentrated and injected onto a HiLoad 26/60 Superdex 200 column that had been pre-equilibrated in buffer C (50 mM HEPES pH 8.0, 50 mM NaCl, and 1 mM TCEP). The column was eluted with 1.5 CV of buffer C. The concentration of FLAG-Parkin was established using the calculated extinction coefficient of 63,440 cm^−1^ M^−1^.

#### R0RBR

Frozen pellets were resuspended in buffer A (50 mM Tris pH 8.0, 200 mM NaCl, 10 mM imidazole, 250 µM TCEP, and EDTA-free Complete protease inhibitor tablets) and lysed using a microfluidizer. The lysate was cleared (45,000 g, 25 min, 4°C) and the supernatant agitated gently with high performance Ni sepharose (0.625 ml resin/L cell culture) for 1 hr at 4°C. The beads were washed with 10 column volumes of buffer A containing 20 mM imidazole and then washed with 10 column volumes of buffer A containing 40 mM imidazole. The R_0_RBR protein was eluted with 10 column volumes of buffer A containing 200 mM imidazole. After elution, the protein was dialyzed into 50 mM Tris for 2 hr at 4 °C to reduce the salt concentration. The protein was then loaded onto a Mono Q HR 10/10 anion exchange column that had been pre-equilibrated in buffer B (50 mM Tris pH 8.0 and 250 µM TCEP). The column was developed with a gradient of 0−500 mM NaCl over 50 column volumes and the protein was eluted at 113−180 mM NaCl. The Sumo tag was then removed by incubation with SENP1 (10∶1 ratio of protein to SENP1) for 2 hr at 4°C. Following the incubation, 10 mM imidazole was added to the cleavage reaction and the reaction was purified over a high performance Ni sepharose column (0.625 ml resin/L cell culture) to remove the Sumo tag and the SENP protease. The Ni column was washed with 10 CV of buffer A. Both the wash and the flow thru from the Ni column were collected and injected onto a HiLoad 26/60 Superdex 200 column that had been pre-equilibrated in buffer C (25 mM HEPES pH 8.0, 50 mM NaCl, and 1 mM TCEP). The column was eluted with 1.5 CV of buffer C. The concentration of R_0_RBR was established using the calculated extinction coefficient of 46,940 cm^−1^ M^−1^.

#### Ubl and Ubch7

Frozen pellets were resuspended in buffer A (50 mM NaHPO4, pH8, 300 mM NaCl, 10mM imidazole, 1% TritonX-100, 2mM β-Mer, 10% glycerol and complete EDTA-free antiproteases) and lysed using a microfluidizer. The soluble fraction was collected after centrifugation at 45,000 g for 30 minutes and purified over nickel sepharose using batch mode 1 hour binding at 4C. The beads were washed with 10 column volumes of buffer A containing 20 mM imidazole and followed by 10 column volumes buffer A containing 40 mM imidazole followed by elution of the proteins with 8 column volumes of buffer A containing 300 mM imidazole. A small fraction of the nickel purified proteins were dialyzed against 50 mM Hepes, pH 8.8, 0.05% Tween-20, 0.01% pluronic F127 and 10% glycerol for Biacore experiments. The concentrations of UBCH7 and UBL were established using the calculated extinction coefficient of 18450 cm−1 M−1 and 11000 cm−1 M−1, respectively. To remove the N-terminal 8xHis tag from Ubch7, the remaining protein was incubated with TEV (10∶1 ratio of protein to TEV) overnight at 4°C via dialysis in buffer A. The cleaved material was purified over a high performance Ni sepharose column to remove the TEV and the tag. The Ni column was washed with 10 CV of buffer A. Both wash and flow through from nickel were collected and injected onto a HiLoad 26/10 Superdex column 200 that was pre-equilibrated in buffer C (50 mM Hepes, pH8, 50 mM NaCl, 1 mM TCEP). The column was eluted in 1.5CV of buffer C.

### Light Scattering Experiments – Proteins

Light scattering experiments were conducted using an Agilent 1100 series HPLC coupled with a Dawn model EOS multiangle light scattering photometer and an Optilab Rex refractive index detector (Wyatt Technology, Santa Barbara, CA). Protein samples (1 mg/ml) were heated at 56°C for 80 min before injecting 100 µl on a Wyatt 30 S guard column followed in series by a Wyatt 30 S column. Experiments were carried out in 50 mM HEPES, 50 mM NaCl, 10% glycerol and either 4 mM dithiothreitol (DTT) or 4 mM TCEP pH 8.0. Size exclusion chromatography was carried out at a flow rate of 0.5 mL/min at room temperature with a run time of 40 min. The experimental data was analyzed using Astra software (Wyatt Technology, Santa Barbara, CA).

### S5a Ubiquitination HTRF assay

FL FLAG-Parkin (35 μM stock) was thermally treated by incubating at 56°C for 30 min. The thermally treated FL FLAG-Parkin stock was stored at −80 C. 5 μl of thermally treated or non-treated FLAG-Parkin diluted in assay buffer was added to wells of a 384-well non-binding plate. 5 μl of a premix of 15 nM E1, 300 nM E2 UbcH7, 1600 nM Ub, 20 nM Ub-Eu K, 200 nM biotinylated-S5a (Boston Biochem), and 1 mM Mg-ATP in assay buffer (50 mM Hepes pH 8.8, 0.005% Tween-20, 0.01% Pluronic F-127 and either 5 mM DTT or TCEP, freshly prepared) was added to each well. The reaction was allowed to proceed for 120 min at 30°C. 10 μl of stop-detection mix was added to a final concentration of 75 nM streptavidin XL665 conjugate, 12 mM EDTA in buffer containing 100 mM Na_2_HPO_4_ pH 7.0, 300 mM KF, and 0.1% BSA. The 20 μl reaction mixture was incubated for 60 min at room temperature. HTRF read using a LJL Analyst plate reader (Molecular Devices) at excitation 320 nm and emission 665 nm & 615 nm.

### Fragment Library

From the in-house screening collection of 25,000 fragments, a subset of 5260 compounds was chosen based on the following criteria: (i) CNS lead-likeness: The compounds selected had low molecular weight, few rings and rotatable bonds, consistent with properties of historic leads that were optimized to drugs (30). In addition, the compounds had low cLogP and low toplogical polar surface area to enhance their potential for high oral bioavailability and CNS penetration [13] (ii) Chemical diversity: We calculated a Unity 2D fingerprint for each compound with Sybyl 8.0 (Tripos International, St. Louis, Missouri). Each compound selected was no closer than a Tanimoto similarity of 0.85 to any other selection [14]. (iii) Solubility: Each compound had solubility >100 μM by light scattering assay (see Text S1). The distributions of physical chemical properties were calculated from the software package ACD/PhysChem Batch (Advanced Chemistry Development, Inc., Toronto, ON, Canada).

For the Negative Control Test Set 38 compounds were chosen from our in-house library of lead-optimized hits and drugs on the market based on the same criteria as for the fragment library.

### Surface Plasmon Resonance (SPR) Experiments

***Fragment screenings*** *and binding level screens of the negative control test set* were performed on a GE Healthcare Biacore 4000 instrument. Anti-FLAG antibody M2 was immobilized on a CM5 sensor chip on spot 1 and 5 of each flow cell using standard amine coupling procedure at a concentration of 100 μg/ml to a level of up to 10 000 response units (RU). Briefly, the carboxyl groups of the sensor surface were activated by injection of a solution containing 0.2 M *N*-ethyl-*N*'-(3-diethylamino-propyl)-carbodiimide and 0.05 M *N*-hydroxysuccinimide. The immobilization procedure was stopped by an injection of 1 M ethanolamine hydrochloride to block remaining ester groups. Spot 3 of each flow cell was activated and deactivated and served as a reference spot for subtraction of non-specific binding data. The promiscuous binding (pb) test proteins, Glutathione-S transferase (GST) and Carbonic Anhydrase II (CA) were amine coupled onto each of the remaining spots of each flow cell to levels of 4500–6000 RU using the same amine coupling method. All immobilization steps were performed at a flow rate of 10 μl/min in HBS-P buffer. FLAG-tagged in-house purified Parkin was captured in 10 mM acetic acid pH 4.5 to levels of up to 6700 RU. The running buffer for fragment screens was 50 mM HEPES pH 7.4, 50 mM NaCl, 4 mM TCEP, 0.005% Tween 20, 0.01% pluronic acid PF127, 2% DMSO.

A 20, 60 or 100 mM DMSO solution of each compound was dispensed directly into 96 or 384 well filter plates, or into a polypropylene storage plate using a Biomek FX or an EDC ATS-100 acoustic dispenser. Plates were sealed or lidded in an appropriate manner. Running buffer was added with a Multidrop instrument to obtain aqueous 25 μM fragment solutions of 2% DMSO. The fragment solutions were then filtered into 96- or 384 well plates with a vacuum filtering device and the plates sealed immediately.

Binding experiments were performed at 25°C by injecting the fragment solutions into the instrument over all flow cells and spots in parallel for 50 sec, with a dissociation time of 60 sec, followed by an extra wash of the flow system with 50% DMSO. The flow rate was 30 μl/min. The time-dependent binding curves were monitored simultaneously on all spots and flow cells. The surfaces were regenerated after six fragment injection cycles by washing the surfaces with an injection of running buffer. The binding levels were determined using the software supplied by the instrument manufacturer. Protein ligands UbcH7 and UblD, previously identified in-house as having a good binding level to non-thermal treated Parkin at low μ-molar affinity, were injected every 20 cycles at 20 μM in subsequent screening to monitor the amount of active Parkin protein during the duration of the screen.

The obtained fragment screening data were solvent corrected, reference subtracted, quality controlled and evaluated using the Biacore 4000 Evaluation Software. Data were exported into Excel and further analyzed as follows: (1) The binding levels of running buffer injections were used to calculate the background noise level (> three-fold σ). (2) All binding level data were calculated by subtracting the background noise level and adjusting them to molecular weight and protein activity levels as measured by the binding level of UblD to Parkin (%Rmax) and (3) assuming linearity between the refractive index change and the molecular weight (MW) of injected fragment, the maximum of binding activity (Rmax) can be calculated by equation 1. R protein is the amount of immobilized or captured protein as measured in response units (RU).
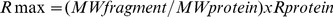
(1)


***Kinetics and Affinity*** measurements were performed on a GE Healthcare Biacore T100 or T200 instrument. Anti-FLAG antibody M2 was immobilized on a CM5 sensor chip using standard amine coupling procedure at a concentration of 100 μg/ml to a level of 10000 response units (RU). Monomeric non-thermal treated FL-Parkin or thermal treated FL-Parkin was captured in 10 mM acetic acid pH 4.5 to levels of up to 16000 RU. The running buffer for both methods was 10 mM HEPES pH 7.4, 150 mM NaCl, 0.005% Tween-20, 4 mM reducing agent. Typically, the 4 flow cells of the sensor chips were used as follows: flow cell 1 served as a reference and was activated and deactivated. Flow cell 2 contained antibody only to control for non-specific binding of fragments to the antibody, and flow cells 3 and 4 contained antibody and captured Parkin. Untagged Parkin domain R0RBR was immobilized by amine coupling at a concentration of 100 μg/ml in 10 mM acetic acid pH 5.0 using the same running buffer as for anti-FLAG antibody immobilization. The surface was then washed with 10 mM HEPES pH 7.4, 150 mM NaCl, 0.005% Tween-20, 4 mM reducing agent for at least one hour.

Kinetics and affinity experiments were performed at 25°C by injecting analyte solutions in two-fold dilutions and at six concentrations from 50 μM for fragments or from a concentration of more than ten-fold above the affinity (K_D_) for protein ligand into the instrument over all flow cells and spots in parallel for 50 sec (fragments) or 120 to 180 sec (protein ligand). During hit confirmation a single injection of 20 μM UblD or UbcH7 was used to monitor Parkin activity. To test binding of reducing agents in the absence of small molecules, the reducing agents were diluted and injected in buffer without reducing agents for 180 sec and a dissociation phase of 600 sec. The flow rate was 30μl/min. The time-dependent binding curves were monitored simultaneously. The surfaces were then regenerated after each binding experiment by washing the surfaces with an injection of running buffer. Kinetics and affinity experiments were repeated at least twice at two different test occasions.

Kinetic and affinity data were solvent corrected (fragments only), reference subtracted and blank subtracted using the Biacore T200 evaluation software V.1. Kinetic constants were determined by curve fitting using a 1∶1 binding model. Association and dissociation curves were fitted globally or locally. The rate of complex formation (1∶1 interaction) during fragment injection was calculated according to the equation (2):
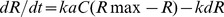
(2)where *R* is the SPR signal in response units (RU), *C* is the concentration of analyte, *R_max_* is the maximum analyte binding capacity in RU, d*R*/d*t* is the rate of SPR signal change. To determine the association constant *k_a_* between fragment and protein, the early binding phase was used. The dissociation phase *k_d_* was measured using the rate of decline in RU after the injection stop, when free running buffer is flowing over the surface. Data were simultaneously fitted by the software and the dissociation constant *K_D_* calculated using equation (3).
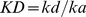
(3)Ligand efficiency (LE, Δg) was calculated as the binding energy of ligand per atom (equation 4)
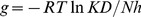
(4)where Nh is the number of heavy (i.e., non-hydrogen) atoms in the ligand.

The dissociation half-life (*t1/2*) was calculated by equation (5).
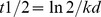
(5)


### NMR

All NMR experiments were conducted at 15°C on a Bruker Avance 700 MHz spectrometer equipped with a 5 mm TCI cryo-probe. 3 mm NMR tubes were used for reducing the protein consumption. 6 µM R0RBR or 4 µM FL FLAG-Parkin protein was diluted in Tris-d11 (Cambridge Isotope) buffer containing 50 mM NaCl at pH 7.5 in >75% D_2_O. The compound concentration was 0.25 mM. Saturation time in the *S*aturation *T*ransfer *D*ifference (STD) experiments was 1.5 sec, and water suppression was used in acquisition. The Mnova 7.0 (Mestrelab Research) was used in data analysis.

## Results and Discussion

Fragment-based Lead Discovery has emerged as one of the most promising strategies in the pharmaceutical industry to identify new leads for their targets [15]. This was made possible by advances in the technological development of NMR and X-ray crystallography. The use of SPR to screen and identify fragment ligands of proteins in drug discovery efforts is a more recent development. However, it has become an increasingly recognized and validated technology for Fragment-Based Lead Discovery [16], [17], [18]. One of its advantages over NMR and X-ray crystallography is the additional kinetic information that it can provide, which can be a valuable tool in structure activity efforts during hit to lead optimization [19]. Fragment-based screening using SPR has been successfully employed on targets such as BACE-1, MMP-12, thrombin and chymase using smaller fragment libraries containing hundreds to thousands of compounds [18].

Previous in-house Parkin HTS screens used a TR-FRET biochemical activity assay, which measured the ubiquitination of the substrate S5a (Rpn10), a subunit of the 19S regulatory complex, which is considered to be a universal substrate to monitor E3 ligase activity [5]. One of the hits from this assay was molecule X (see Figure S1), which had a high nano-molar EC50 in the TR-FRET S5a assay. Its affinity as measured by SPR was also high nano-molar and it served as a tool compound in a variety of assays. The potencies and residency times for the scaffolds that were identified by the TR-FRET activity assay screen proved difficult to optimize using traditional medicinal chemistry efforts and meaningful SAR was not observed. A plot of the off-rate *versus* on-rate, as measured by SPR (Figure S2), shows no improvement in affinity below 100nM and off-rates were not slowed as desired. This was partially due to the result of poor solubility of these scaffolds (2–5 μM). Furthermore, zinc binding was observed for some of these scaffolds. To find novel scaffolds, SPR was used as a tool to define functionally active Parkin, to design and optimize a Parkin Fragment Screen, and to function as the primary fragment screening technology. Compounds scored as hits in the primary screen were then confirmed in dose responses by SPR, NMR and X-Ray Crystallography.

### The Reducing Agent DTT binds to Parkin

**Figure 2:**
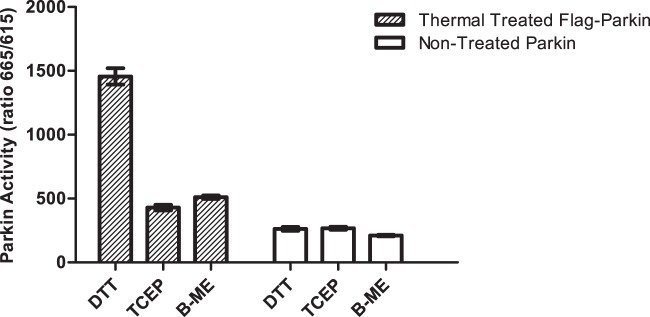
Thermal treated FL-FLAG-Parkin is more active than non-thermal treated FL-FLAG-Parkin independent of reducing agent. TR-FRET S5a assay using 150 nM FL-FLAG Parkin and 200nM biotinylated S5a substrate in the presence or absence of 5 mM reducing agent. FL-FLAG Parkin was incubated at 56°C for 30 min and then cooled to RT (thermal treated). Thermal treated FL-FLAG Parkin exhibit significantly different levels of Parkin activity in presence of each of the three reducing agents p<0.0001 (hatched bar) (n = 4). Non thermal treated FL-FLAG Parkin showed similar levels of activity in presence of either DTT or TCEP with p = 0.5368 and both activities are significantly higher than in presence of BME with p<0.0001 (white bar) (n = 4).

Since Parkin contains a large number of zinc atoms (eight) that are coordinated by 35 cysteines, the choice of a reducing agent for an SPR screen is of high relevance. To measure Parkin ubiquitination activity, the S5a TR FRET biochemical assay was used to test a variety of different reducing agents for their effect on Parkin activity. FL-FLAG-Parkin is heated to 56°C, close to its T*_m_* of 55°C (data not shown) before testing ubiquitination activity. In the presence of a compound that binds Parkin and confers stabilization energy, the Tm of Parkin will shift. Thus, after cooling, Parkin heated in the presence of compound would be expected to have greater activity compared to Parkin heated without compound. Higher affinity compounds would be expected to confer greater stability and preserve more Parkin activity. FL-FLAG Parkin does retain some activity after heating (Figure 2) and the assay can be used to find activators or stabilizers of Parkin if performed in presence of compound. Figure 2 shows that heating Parkin in the presence of different reducing agents increased substrate S5a ubiquitination activity as compared to non-thermal treated Parkin. The increase is dependent on the type of reducing agent: at the optimal concentration of 5–10 mM of DTT, Parkin activity was improved by 6 to 8-fold, while β-mercaptoethanol (BME) and TCEP improved activity by only up to 2.5–fold (Figure 2). To test the dependence on reducing agent in SPR assays, binding activities of the previously identified tool compound X (see Figure S1) was measured in presence of the different reducing agents, respectively. No binding of compound X to thermal-treated or non-treated FL-FLAG Parkin was observed in the presence of BME or TCEP or in the absence of DTT by SPR. However, binding activity of compound X in the presence of DTT in the running buffer and the analyte injections was observed for Parkin that was purified in presence of either BME or TCEP. Therefore, it can be concluded that DTT is required for the binding of compound X to thermal-treated or non-treated FL-FLAG Parkin.

**Figure 3:**
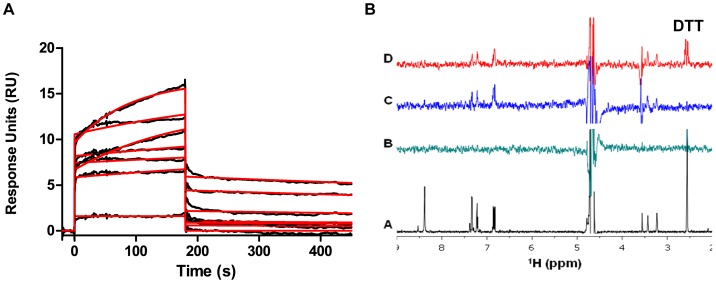
Dithiothreitol (DTT) binds to non-thermal treated FL-FLAG Parkin. (A) SPR data: DTT was injected at 62.5, 31.25, 15.62, 7.8, 3.9, 1.95 and 0.98, 1.9 μM in 50 mM HEPES pH 8.8, 0.005% Tween-20, 0.01% PF-127. The kinetic fits are shown in red. The affinity was determined to 1.4 μM at 47% Rmax (Rmax: 34 RU). DTT dissociates very slowly and the sensorgrams show ill-behavior above 16.5 μM. SPR data for thermal treated FL-FLAG Parkin was similar (data not shown) (B) NMR-STD: (a) Proton spectrum of 0.25 mM compound Z (black); (b) STD spectrum of 0.25 mM compound Z (green); (c) STD spectrum of 0.25 mM compound Z in the presence of 4 µM FL Parkin protein (blue); (d) STD spectrum of 0.25 mM compound Z in the presence of 4 µM FL Parkin protein and 0.5 mM DTT (red).

To mechanistically understand these results, all three reducing agents were tested for direct binding to non-thermal and thermal-treated Parkin using SPR. TCEP and BME did not exhibit any binding to Parkin (data not shown). In contrast, DTT showed binding to both Parkin preparations consistent with a 1∶1 binding mode with low micro-molar affinity and with high binding activity (%Rmax) (Figure 3A). Furthermore, DTT dissociated slowly from Parkin. This can be seen in the dissociation phase of the sensorgrams where the lines are not returning to zero before the next injection starts. A half-life of 28.95 min was calculated from the dissociation rate k_d_. The sensorgrams are typical for a 1∶1 binding event but showed some ill-behavior at concentrations above 16.5 μM DTT, reminiscent of either a possible secondary binding-site or a conformational change of Parkin protein. Furthermore, DTT was tested for binding to FL-FLAG-Parkin by NMR in a Saturation Transfer Difference experiment (Figure 3B). DTT binding was evident in the STD spectrum of compound Z in the presence of Parkin protein and DTT at 2.6 ppm of the proton spectrum. In summary, DTT was shown to bind to Parkin protein by both SPR and NMR. Because DTT binding could potentially change the conformation of Parkin or chelate zinc, DTT was omitted from the SPR Parkin screening buffer.

### Parkin on the sensor chip is an oligomer with functional activity

In order to reduce non-specific binding of fragments to Parkin during the fragment screen, it is very important to confirm the functional activity and correct folding of the protein on the sensor chip [20]. This was accomplished by testing different capture antibody densities and Parkin capture levels on the sensor chip for binding of different reference compounds and protein ligand.

Non-thermal treated FL-FLAG Parkin purified from insect cells was monomeric and pure. A single peak was observed in SEC and elution fractions of a SEC column did run at its expected MW in SDS-PAGE gel (see Figure S3). DLS and MALS data also confirm a MW correlating to a monomeric state (Table S1).

To capture FL-FLAG-Parkin on a sensor chip, anti-FLAG antibody was immobilized by amine coupling and then FL-FLAG Parkin purified in the presence of TCEP was injected. Several different densities of anti-FLAG Ab were tested for Parkin capture. However, if the capture reaction was allowed to run until completion, FL-FLAG Parkin consistently captured at a stoichiometry of 3∶1 (Parkin: Ab) independent of the density of capturing anti-FLAG antibody immobilized or if FL- Parkin protein was thermal treated or not (data not shown). This 3∶1 stoichiometry was also required to achieve the highest % Rmax for small molecule binders identified from the previous Parkin HTS screen such as compound X.

Thermal treated Parkin was observed to be oligomeric with a molecular weight range of between 200 and 800 kDa using gel filtration (Table S1). This finding was also supported by Multi Angle Light Scattering (MALS) data as well as DLS data (see Table S1). Thermal treated FL-FLAG Parkin was more active in the enzymatic activity assay S5a than non-thermal treated Parkin (see above). The observation that oligomeric E3 ligases are functionally active has also been reported in the literature: For example, Ring dimerization is a prerequisite for the formation of an active E3 ubiquitin ligase [21], Ring domains of unrelated proteins can self-assemble and subsequently form nanobodies [22], [23]. Furthermore, it has been speculated that this supra-molecular nature of Ring domains is required for enhancement of activity.

**Figure 4:**
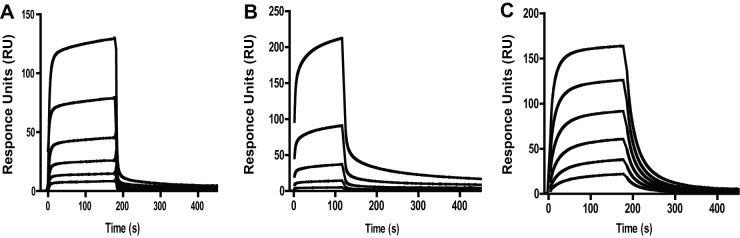
Functionally active FL-Parkin binds three different protein ligands. FL-FLAG Parkin was captured on a CM5 sensor chip with immobilized anti-FLAG antibody at a stoichiometry of (3∶1) (Parkin:Ab). Each of the protein ligands was injected at concentrations above 10-fold K_D_ if possible: (a) Ubiquitin at 500, 250, 125, 62.5, 31.25 and 15.62 μM (b) His-UblD and (c) UbcH7 were injected at 140, 46.7, 15.6, 5.2, 1.7 and 0.6 μM over FL-FLAG Parkin. All data was fitted to 1∶1 binding model. Each binding test was repeated at different test occasions (n≥3).1∶1 binding isotherms are shown in Figure S4–6.

To confirm that FL-FLAG Parkin captured at a 3∶1 stoichiometry is functionally active, binding of three protein ligands that are reported in the literature to bind to Parkin were tested by SPR. These three ligands were ubiquitin [10], the ubiquitin-like domain of Parkin (UblD) [9] and the E2 ubiquitin conjugating enzyme, UbcH7 [23]. SPR confirmed binding of these three proteins to FL-FLAG Parkin and SPR was also used to determine their respective binding affinities (Table 1 and Figure 4):

**Table 1:** Affinities K_D_ (μM) of protein ligands to different Parkin proteins.

Protein/Ligand	UblD^1^	UbcH7^2^	Ubq^3^
**FL^4^-FLAG-Parkin RT**	5.4	4.7	82
**FL^4^-FLAG-Parkin 56°C**	7.4	8.1	n/a
**R0RBR Domain RT**	6	7.1	96

1 **Ubiquitin-like domain of Parkin; ^2^Ubiquitin conjugating enzyme E2; ^3^Ubiquitin; ^4^ Full-length.**

(i) Ubiquitin bound to FL-FLAG Parkin by SPR at an affinity of 82 μM, which agrees well with the previously reported value of high micro-molar affinities for mono-ubiquitination of Ubiquitin binding domains [24] (Figure 4A and S4). (ii) His-tagged UblD (Figure 4B and S5) was fitted to a 1:1 binding model and exhibited an affinity (K_D_) of 5.4 μM for non thermal treated and 7.4 μM for thermal treated Parkin. The binding affinity for the Parkin domain R0RBR was determined to be 6 μM. The affinity data as determined by SPR agrees well with a reported affinity of 2.6 μM (K_D_) and one binding site for binding of the UblD domain to ΔUblD-Parkin in the literature [9].

(iii)The E2 ubiquitin conjugating enzyme UbcH7 is required for the enzymatic activity of Parkin [3]. Activated Ubiquitin is transferred to the UbcH7 from the E1 Activating enzyme. Ubq-E2 then binds to Parkin and Ubq is conjugated to the substrate. Binding of UbcH7 is supported by X-ray data that show binding of UbcH7 to the E3 ligase c-Cbl in presence of phosphorylated peptide ZAP-70 [25]. Furthermore, UbcH7 binding to Parkin was inferred by its activity in ubiquitination assays [23]. Non-tagged UbcH7 was purified in house and tested for binding to FL-FLAG Parkin at concentrations between 50 μM and 0.5 μM in two-fold dilutions, as done for UblD (Figure 4C and S6). An affinity of 4.7 μM was determined for non-thermal treated Parkin to UbcH7, 8.1 μM for thermal treated FL-FLAG Parkin to UbcH7, and 7.1 μM for the Parkin domain R0RBR to UbcH7

Taken together, the SPR binding affinity data of Ubq, UblD and Ubch7 for Parkin shows that FL-FLAG Parkin captured by an anti-FLAG antibody on a sensor chip in a 3∶1 stoichiometry binds protein ligands as reported in the literature and with similar affinities as determined by solution phase methods (Ubq and UblD). This suggests that Parkin captured on a Biacore chip is suited for screening purposes. As protein ligand binding was independent of Parkin thermal treatment, non-thermal treated FL-FLAG Parkin was selected for the fragment screen. The binding affinities of UblD and UbcH7 to the Parkin domain R0RBR were also very similar to FL-Parkin. This would suggest that the R0RBR domain is properly folded and is relevant for use in NMR experiments (see below).

As both UblD and UbcH7 dissociated at a fast rate (*k_d_*) (2 min for UblD and 30 sec for UbcH7), injections of these protein ligands followed by buffer injections were used throughout the fragment screen to monitor the extent of Parkin activity during a screening run. Ubiquitin binding was not used as a positive control for Parkin activity as the affinity was quite weak (high micro-molar range). The two reference proteins bound only to Parkin protein and not to control proteins such as CAII and GST (data not shown).

### Buffer Optimization

Parkin has 35 cysteines, 8 zinc ions and exhibits a tendency for aggregation and oligomerization. It was therefore of great importance for the Parkin SPR Fragment Screen to use a buffer that was optimized for salt and detergent composition, pH and DMSO content to maximize Parkin stability during a screening run. Furthermore, no small molecule is known in the literature to bind to Parkin that would have been suitable as a positive control. Therefore, different buffer conditions were tested to minimize non-specific binding of a Negative Control Test Set of 38 compounds to FL-FLAG Parkin by SPR. This set of compounds was designed to have good Lipinski properties, be diverse with respect to chemical space and therapeutic area. Furthermore, compounds were not expected to bind to Parkin, because of their affinity for targets not related to E3 ligases. For each condition, Parkin's functional activity was tested by injecting the known protein ligands UblD and UbcH7. The buffer composition exhibiting the lowest number of Negative Test Set compounds binding while retaining acceptable Parkin functional activity was then used for the Parkin Fragment SPR Screen. Different detergents, pH ranges from 7.0 to 8.8, DMSO percentages from 0.5 to 5%, salt concentrations from 0 to 150 mM, and BME and TCEP from 1 to 4 mM were tested for binding of the Negative Test Set using the Biacore 4000 (data not shown). Tween-20 or pluronic F-127 detergent or a combination of both did not have any influence on Parkin activity or compound binding. Optimal buffer conditions were found at 2% DMSO and 50 mM NaCl as protein activity decreased dramatically to 50% at DMSO concentrations greater than 2.5% and NaCl concentrations greater than 100 mM. Furthermore, the number of negative test set binders decreased from two binders at no salt to zero binders at 50 mM NaCl. There was no difference in negative control test set binding noted if either TCEP or BME was used. With reducing agent concentration below 4 mM the activity of Parkin decreased more rapidly and was not high enough for the duration of a screen. The optimal buffer composition was determined to be 50 mM HEPES pH 7.4, 50 mM NaCl, 4 mM TCEP, 0.005% Tween 20, 0.01% pluronic acid PF127 and was confirmed in a mock run using the Negative Test Set compounds as analytes and reference proteins UblD and UbcH7 to confirm functional activity of non thermal treated FL-Parkin.

### Parkin Fragment Screen

FL-FLAG Parkin was captured at 16000 RU levels at a stoichiometry of 3∶1 (Parkin:Antibody) to yield a %Rmax of 60–70 RU for the majority of fragments included in the screen. To measure promiscuous off-target binding, proteins were chosen with features similar to Parkin. Carbonic Anhydrase II, which contains one Zn coordinated to three histidyl residues and one water molecule [26], was chosen to serve as indicator for possible Zn binding compounds. Human Glutathione-S transferase (GST) was included as it is a dimer [27]. Both proteins were immobilized at RU levels that would yield a %Rmax for fragment binding similar to FL-FLAG –Parkin.

Solubility is a major contributor to the ill-behavior of compounds in a SPR experiment. This can be readily observed in their SPR sensorgrams; for example those that exhibit slow upwards drift during injection which is reminiscent of non-specific binding, or carry over or slow non 1∶1 dissociations after injections [28]. Therefore, all compounds included in the screen were first tested for solubility, and a cut-off of 100 μM was set as acceptable. Furthermore, SPR as a primary screening tool is most suitable for fragments with an affinity of 1 mM or better [29], although fragments with an affinity of 5 mM have been identified [20]. In order to minimize solubility issues and to find binders with affinities of at least high micro-molar range, fragments were injected at 25 μM. The theoretical lowest affinity of a 150 Da fragment was calculated as described by Navratilova [18] and was determined to be 0.13 mM even if only 50% of the protein were found to be active, which was a reasonable cut off.

**Figure 5:**
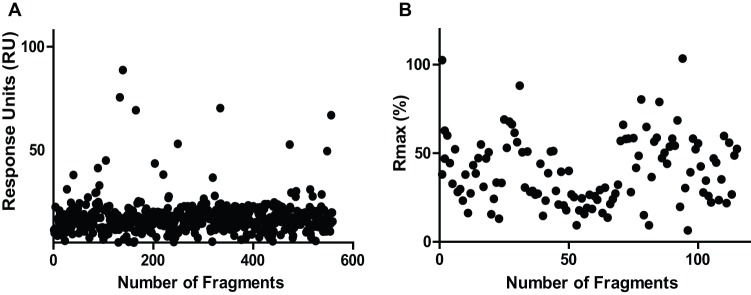
Parkin fragment screen data. (A) Graphical representation of a typical run of the Parkin fragment screen representing 10% of the total number of fragments screened (560 fragments). The binding level of each fragment of the run is shown in Response Units (RU) on the x-axis and the number of fragments on the y-axis. Experiments were performed on a Biacore 4000 instrument w buffer containing 2% DMSO. FL-FLAG Parkin was captured on a CM5 sensor chip with immobilized anti-FLAG antibody at a stoichiometry of (3∶1) (Parkin:Ab). Fragments were injected at 25 μM in buffer containing 2% DMSO All data were reference subtracted, solvent corrected and adjusted for changes in surface activity during a run.(B) Fragment binding levels as %Rmax of single concentration SPR hits at 25 uM of a screen of 5260 fragments. Only hits with a binding level greater than 3-fold standard deviation (SD) and acceptable sensorgrams are shown.

5260 fragments were injected at 25 μM. The data was solvent corrected and reference subtracted. The background noise level was calculated as outlined in Materials and Methods. A typical single run of the fragment screen is shown in Figure 5A. Every hit above this level was visually inspected for ill-behavior from its individual sensorgram. Only fragments without ill-behavior and without superstoichiometry were included in the hit list. A hit rate of 2.14% was obtained. This is a low hit rate in comparison to other single step fragment screens, in which the usual hit rates are between 5 to 15% depending on the screening concentration, target protein and its activity [20]. Throughout each screening run binding levels of the two reference protein ligands, UblD and UbcH7 decreased by only 15%, confirming a high functional activity level of Parkin throughout the entire screen. The low hit rate likely resulted from the fragment concentration and the extensive buffer optimization with the Negative Test Set of compounds. Figure 5B shows graphically all 117 Parkin binders as %Rmax above the background noise (see Materials and Methods). The majority of hits had a %Rmax of 20–50, and only one fragment bound to CAII and three fragments to GST. This low promiscuous binding rate (0.08%) is possibly another positive effect of the buffer optimization with the Negative Test Set.

### Confirmation of single concentration SPR Fragment Hits

**Figure 6:**
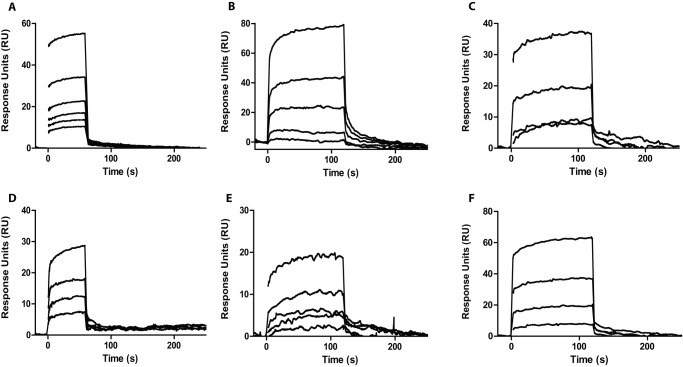
Confirmation of fragment single concentration hits in dose response manner. FL-FLAG Parkin was captured on a CM5 sensor chip with immobilized anti-FLAG antibody at a stoichiometry of (3∶1) (Parkin:Ab). Fragments were injected at 50, 25, 12.5, 6.25, 3.1 and 1.5 μM. All SPR Fragment hits were confirmed by NMR; SPR Fragment hit I was also confirmed by X-ray crystallography. All data was fitted to 1∶1 binding model. Each binding test was repeated at different test occasions (n≥2).

In order to confirm the activity of the SPR fragment hits that were obtained at a single concentration, they were tested for binding to the target protein in dose response format, with a starting concentration of 50 μM followed by two-fold dilutions downward. A confirmed hit was defined as fragment binding in a concentration-dependent manner at equal or more than three concentrations. All single concentration hits were confirmed again for at least one concentration, and 36 fragments were confirmed to have a dose response. 15% of these fragments had weak affinities, with a calculated % Rmax below 10% at the concentrations tested. These fragments were therefore not further pursued. Concentrations higher than 50 μM were not tested to avoid loss of compound solubility, which could interfere with a good SPR signal. Another 38% of the confirmed hits showed ill-behavior at concentrations above 25 μM. 13 hits showed concentration-dependent binding and displayed sensorgrams without any sign of ill-behavior. The result was a 0.21% overall hit rate as calculated from the total number of fragments screened. These 13 confirmed hits and some of the single concentration hits with promising sensorgram shape were tested for binding to the R0RBR domain of Parkin by STD NMR. Overall, 16 fragments were confirmed as binders to Parkin by NMR. These comprised 12 of the confirmed hits and 4 of the single concentration hits. Taken together, good agreement between SPR and NMR binding data was achieved as 16 out of the 21 fragments tested by NMR bound to the Parkin R0RBR domain. The SPR screen used FL-FLAG –Parkin, while the NMR screen used the parkin R0RBR domain. It is, therefore, possible that the SPR hits that were negative in binding to R0RBR by NMR bind to a region outside of this domain. Figure 6 shows some examples of SPR hits that were confirmed by NMR and or X-ray crystallography. Overall, sensorgrams exhibited traces that showed no signs of aggregation, ill-behavior or solubility issues.

**Figure 7:**
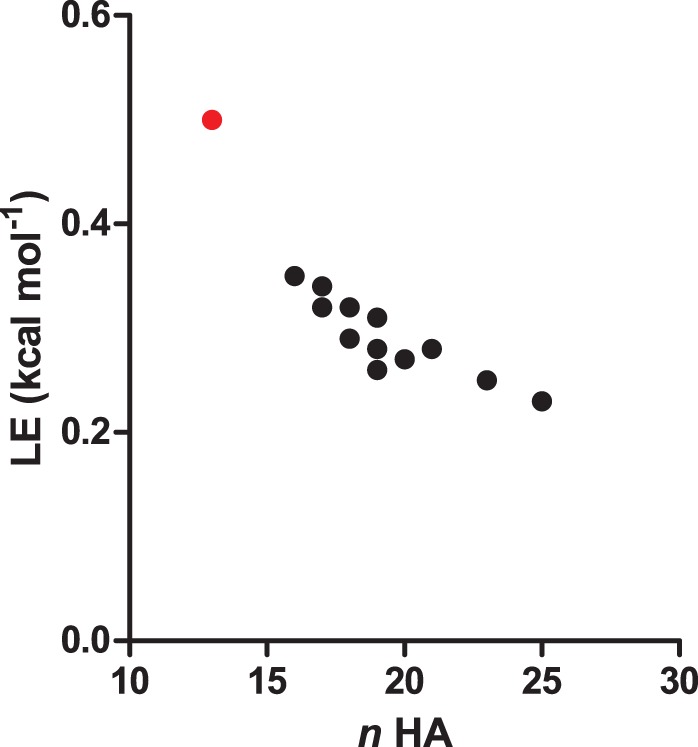
Ligand efficiencies of confirmed SPR hits. Plot of ligand efficiencies (LE) of SPR confirmed hits vs. number of heavy atoms *N_h._* Binding affinities (K_D_) were obtained from injection of fragments in dose response manner from 50 uM in two-fold dilutions. One very interesting hit with LE of 0.5 and *HAC* of 13 is A (red dot) with a mid micro-molar affinity (K_D_ of 18.6 uM).

The data obtained from dose response confirmation assay were also used to calculate the affinity of the 13 confirmed hits (see Table 1). The affinities (K_D_) ranged from roughly 20 μM to 220 μM. This shows that fragments with an approximately ten-fold higher K_D_ than the ligand concentration of the single concentration screen were detected. This confirms that fragment hits were detected above the theoretical K_D_ of 0.13 mM. This is probably due to the high functional activity of Parkin throughout the screen. For each fragment hit confirmed by dose response, ligand efficiency (LE) was calculated using the Heavy Atom Count (HAC) (Table 2). A plot of LE vs. HAC (Figure 7) shows a distribution that is consistent with those reported in the literature [30]. Fragments with affinities above 100 μM also have a number of heavy atoms above 17 and up to 19. One very interesting hit with LE of 0.5 and *n*_HA_ of 13 is Fragment A with a K_D_ of 18.6 μM. This hit was also confirmed as a binder by NMR and is of interest for hit to lead expansion.

**Table 2:** Fragment hits confirmed in dose response and their affinities K_D_ (μM) for FL-Parkin as determined by SPR as well confirmation by NMR.

Fragment	*N_h_*	K_D_ (μM)	LE (kcal/mol)	NMR confirmed
**A**	13	18.3	0.50	yes
**B**	16	68.2	0.35	no
**C**	17	110	0.32	yes
**D**	17	50.3	0.34	yes
**E**	18	67	0.32	yes
**F**	18	128	0.29	yes
**G**	19	50	0.31	no
**H**	19	111	0.28	yes
**I**	19	220	0.26	yes
**J**	20	120	0.27	yes
**K**	21	53.4	0.28	yes
**L**	23	70	0.25	yes
**M**	25	55	0.23	yes

SPR Affinity (K_D_) data and the number of heavy atoms *N_h_* was used to calculate ligand efficiency (LE) data.

### Physical-Chemical Characteristics of SPR Fragment Hits

**Figure 8:**
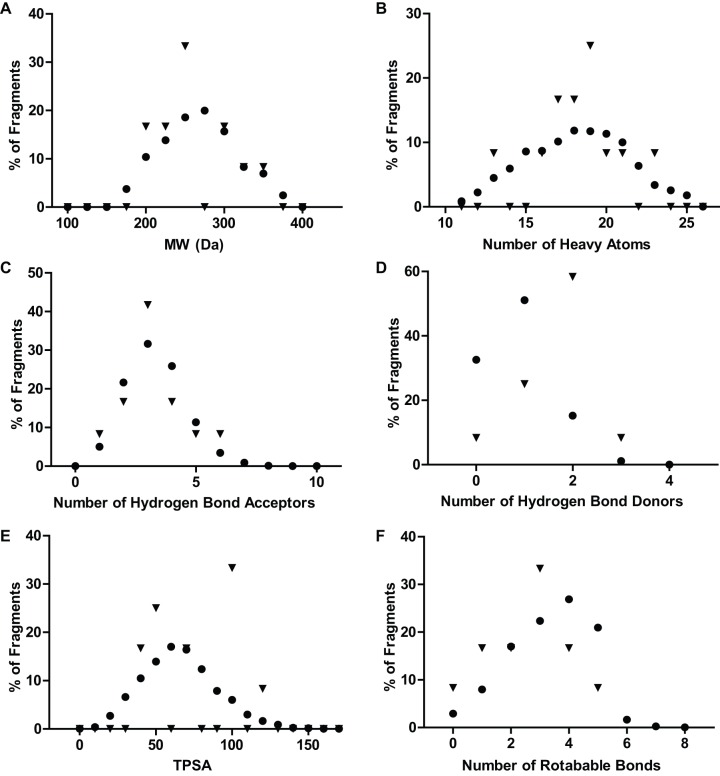
Physico-chemical properties of SPR hits vs. fragment library. The distribution of compounds in the fragment library is shown as circles; the SPR hits as triangles. The distribution of SPR hits is consistent with the fragment library (though noisier because of the small number of compounds), with the exception of hydrogen bond donors, which are over-represented in the SPR hits compared to fragment library.

To further characterize the SPR fragment hits, physical-chemical properties of the fragment library were plotted versus fragment hits by both SPR and NMR. The distributions of physical chemical properties for the fragment library are shown in Figure 8. These distributions are consistent with fragment libraries reported in the literature [15], [31]–[32]. For example, the distribution of calculated log P is between 0 and +3 and does not differ between the fragment library and the SPR hits (data not shown). Also the average molecular weight distribution is a relatively low at 250 Daltons as are the counts for hydrogen bond donors and acceptors. The topological polar surface area (TPSA) is shifted to lower values, which reflects the design criterion to increase the likelihood of crossing the blood brain barrier for CNS-related targets. The comparison of distributions of the physical-chemical characteristics of the SPR hits in the fragment library showed that greater than 40% had 3 Hydrogen Bond Acceptors, 60% had 2 Hydrogen Bond Donors, 30% of the hits had a MW of 250 Daltons and 25% had 19 heavy atoms. These values are statistically significant and will be useful to characterize the binding pocket(s) for these hits and give guidance for hit to lead expansion by SAR. Although a number of distinct chemotypes are represented in the active fragments, quinoline and thiazole moieties are present in multiple compounds. This serves as a consistency check and could also form the basis for nascent structure-activity relationships for these scaffolds.

In summary, SPR was used to define active Parkin and optimize screening conditions. 5260 fragments were screened at a single concentration in a primary SPR screen. Very low binding levels were found to promiscuous binding proteins. The overall hit rate was low, but hits were of medium affinity, ranging from 20 to 220 μM. A high correlation between SPR and NMR of ∼80% was obtained. Confirmed hits exhibited lead-like quality in their sensorgrams, have reasonable Lipinski properties, and are therefore excellent candidates for hit to lead expansion.

## Supporting Information

Figure S1 Lead-optimized small molecule X from a previous Parkin screen binds to Parkin at an affinity of 200nM as measured by SPR. FL**-**FLAG Parkin was captured on a CM5 sensor chip with immobilized anti-FLAG antibody at a stoichiometry of (3∶1) (Parkin:Ab). Fragments were injected at 500 nM and down to 15.6 nM in two-fold dilutions in a buffer containing 50 mM HEPES pH 8.8, 4 mM DTT, 0.005% Tween-20, 0.01% pluronic acid PF127. Small molecule X is marked by a black arrow. Kinetic data were calculated by fitting to a 1∶1 binding model. Binding tests were repeated at different test occasions (n = 3).
(EPS)

Figure S2 Previous Parkin Screen hit expansion data. On-rate/off-rate heat map of previous hit to lead optimized Parkin hits. Compound X at 200 nM is marked. At least 6 concentrations of small molecules were injected from 5 μM down to 0.15 μM in two-fold dilutions. Kinetic data were calculated by fitting to a 1∶1 binding model. Binding tests were repeated at different test occasions (n = 3).
(EPS)

Figure S3 N-terminally FLAG-tagged Parkin expressed in insect cells is monomeric. Size exclusion chromatography (SEC) elution profile of FL-FLAG Parkin (A)SDS PAGE analysis of the SEC fractions (B). The predicted MW is 55 kD.
(EPS)

Figure S4 1∶1 Binding isotherms of Ubiquitin to Parkin. FL-FLAG Parkin was captured on a CM5 sensor chip with immobilized anti-FLAG antibody at a stoichiometry of (3∶1) (Parkin:Ab). The protein ligand was injected at concentrations above 10-fold K_D_ if possible: Ubiquitin at 500, 250, 125, 62.5, 31.25 and 15.62 μM over FL-FLAG Parkin. All data was fitted to 1∶1 binding model. Each binding test was repeated at different test occasions (n≥3).
(EPS)

Figure S5 1∶1 Binding isotherms of His-UblD to Parkin. FL-FLAG Parkin was captured on a CM5 sensor chip with immobilized anti-FLAG antibody at a stoichiometry of (3∶1) (Parkin:Ab). The protein ligand was injected at concentrations above 10-fold K_D_ if possible: His-UblD was injected at 140, 46.7, 15.6, 5.2, 1.7 and 0.6 μM over FL-FLAG Parkin. All data was fitted to 1∶1 binding model. Each binding test was repeated at different test occasions (n≥3).
(EPS)

Figure S6 1∶1 Binding isotherms of UbcH7 to Parkin. FL-FLAG Parkin was captured on a CM5 sensor chip with immobilized anti-FLAG antibody at a stoichiometry of (3∶1) (Parkin:Ab). The protein ligand was injected at concentrations above 10-fold K_D_ if possible: UbcH7 was injected at 140, 46.7, 15.6, 5.2, 1.7 and 0.6 μM over FL-FLAG Parkin. All data was fitted to 1∶1 binding model. Each binding test was repeated at different test occasions (n≥3).
(EPS)

Table S1 Thermal treated FL-FLAG Parkin has a larger molecular weight consistent with oligomerization. A. Dynamic Light Scattering data; B. SEC-MALS data.
(DOCX)

Text S1 (DOCX)
