# Correction: Label Free Fragment Screening Using Surface Plasmon Resonance as a Tool for Fragment Finding – Analyzing Parkin, a Difficult CNS Target

**DOI:** 10.1371/annotation/590bbe38-3a12-45bd-bc5d-83b766c968b3

**Published:** 2013-07-30

**Authors:** Karin Regnström, Jiangli Yan, Lan Nguyen, Kari Callaway, Yanli Yang, Linnea Diep, Weimei Xing, Anirban Adhikari, Paul Beroza, Roy K. Hom, Brigit Riley, Don Rudolph, Michael F. Jobling, Jeanne Baker, Jennifer Johnston, Andrei Konradi, Michael P. Bova, Dean R. Artis

The name of the last author is incorrect. The correct name is: Dean R. Artis. The correct abbreviation in Contributions is: DRA. 

