# Supplementary material for: Label Free Fragment Screening Using Surface Plasmon Resonance as a Tool for Fragment Finding – Analyzing Parkin, a Difficult CNS Target
Source: PLoS One. 2013 Jul 5;8(7):e66879. doi: 10.1371/journal.pone.0066879 (PMC3702509; doi:10.1371/journal.pone.0066879)
Supplement: Table S1 — Thermal treated FL-FLAG Parkin has a larger molecular weight consistent with oligomerization. A. Dynamic Light Scattering data; B. SEC-MALS data. (DOCX) [file pone.0066879.s007.docx]

**Table S1:**

**A.**

| **Protein** | **R (nm)** | **% Pd** | **MW (kDa)** | **% Intensity** | **% Mass** |
| --- | --- | --- | --- | --- | --- |
| **FL-FLAG-Parkin** | 3.7 | 23.5 | 70.8 | 93.2 | 99.9 |
| **Thermal-treated FL-FLAG Parkin** | 7.4 | 19 | 357.6 | 100 | 100 |

**B.**

| **Protein** | **MW (kDa)** | **MW Range (kDa)** |
| --- | --- | --- |
| **FL-FLAG-Parkin** | 66.9 | 56-80 |
| **Thermal-treated FL-FLAG Parkin** | 310.8 | 200-800 |
