# Supplementary material for: Label Free Fragment Screening Using Surface Plasmon Resonance as a Tool for Fragment Finding – Analyzing Parkin, a Difficult CNS Target
Source: PLoS One. 2013 Jul 5;8(7):e66879. doi: 10.1371/journal.pone.0066879 (PMC3702509; doi:10.1371/journal.pone.0066879)
Supplement: Text S1 — (DOCX) [file pone.0066879.s008.docx]

**Text S1**

Compound solubility was measured by forward light scattering using a BMG Nephelostar plate reader. Briefly, each compound was tested at 500uM and 100uM (5% DMSO) in the Parkin AlphaScreen assay buffer, and following a 60 minute incubation read at 635nm. Each compound was then placed into one of three bins for solubility rating purposes; 1. 505 uM solubility – soluble at >500 uM (high solubility); 2. 300 uM solubility – soluble at >100 uM, but showing insolubility at 500 uM (moderate solubility); 3. 95 uM solubility – showing insolubility at 100 uM (poor solubility).
